# Unqualified Medical "Assistants"

**Published:** 1897-12-04

**Authors:** 


### The Hospital

A Journal of

The Medical Sciences and Hospital Administration.

Vol. XXIII.—No. 584.] Edited by Sir Henry Burdett, K.C.B., and Solomon C. Smith, M.D., M.R.C.P. [Dec. 4, 1897.

### Unqualified Medical "Assistants."

The General Medical Council, in the course of the session just concluded, has taken the final step, long in contemplation, of absolutely prohibiting the employment by medical men of unqualified so-called "assistants" in the treatment of patients, and has resolved to send to every practitioner upon the Register, and to give, at the time of registration, to every newly-qualified person, the following notice :—

"Whereas it has from time to time been made to appear to the General Medical Council that some registered medical practitioners have been in the habit of employing as assistants, in connection with their professional practice, persons who are not duly qualified or registered under the Medical Acts, and have knowingly allowed such unqualified persons to attend or treat patients in respect of matters requiring professional discretion or skill: and whereas in the opinion of the Council such a substitution of the services of an unqualified person for those of a registered medical practitioner is in its nature fraudulent and dangerous to the public health: the Council hereby give notice that any registered medical practitioner, who is proved to have so employed an unqualified assistant, is liable to be judged as guilty of 'infamous conduct in a professional respect,' and to have his name erased from the Medical Register under the 29th Section of the Medical Act, 1858."

The "unqualified assistant," taking him as an individual, is sometimes a person who seems not to be altogether undeserving of sympathy, but, taking him in the aggregate, he constitutes, and for several years past has constituted, a grievous disgrace and scandal. In many instances he is the son of a medical practitioner, and, after receiving a scanty school education, has commenced his career as a medical student. This career has been interrupted, sometimes by failure of means, sometimes by inability to pass an examination. When a student is deprived of means by misfortune, and is a man of character and energy, a helping hand is usually extended to him by friends; and, although he may be compelled to devote a portion of his time to earning money, he is often enabled to do this in such a manner as to leave him a sufficient margin for study, and for the attainment of the knowledge necessary for qualification. When he possesses neither energy nor character, he usually places the coping-stone upon his unfitness by marrying a penniless girl, and by undertaking to maintain a wife and possible children before he has any assured means of maintaining himself. He then advertises in the medical journals for a situation, describing himself, it may be, as "a fourth year's student, desirous, &c., &c.," and his advertisement is answered by some doctor in large and widely-spread practice among the working classes, perhaps in some colliery or mining district, perhaps in the poorer portion of some populous town. The advertiser, in such a case, declares that it is his intention to obtain a qualification in the course of a few months, and the employer promises him time to read; each party to the bargain having his tongue in his cheek. The "reading" will probably be restricted to a sporting newspaper or to *Tit-Bits*, and the assistant, as time goes on, will rest his claim to the continued confidence of the public and of his employer mainly upon his "experience." It is a maxim of political economy that every learner spoils a portion of raw material; and the experience of the unqualified assistant is obtained at the cost of health or life to many of those who are so unfortunate as to fall under his ministrations. But he will be hail-fellow-well-met with the class of people whom he attends, and will often be popular among them. By the time he is forty years old he will probably assume the title of "Doctor" and will set up for himself, perhaps hiring some young man just passed, and of character kindred to his own, to "cover" him by signing death certificates for his patients. In view of these considerations, but in view also of the existence of a certain number of exceptions to the general rule, exceptions deserving of favourable consideration, the Medical Council has hitherto been content to say that the system was doomed, and that the employment of unqualified assistants to attend the sick would eventually be prohibited, thus affording an ample margin of time in which those who were fit to do so might place themselves upon the Register. The period of grace has now expired, and, just as no one can officiate as a clergyman until he is in orders, or as a solicitor until he has passed the prescribed examinations, so, for the future, no one will be permitted to attend patients as an assistant until he has obtained a qualification. The unqualified man who practises medicine, surgery, or midwifery will have to do so independently, and as an avowed quack; the fraud of passing him off upon patients as a person worthy of their confidence being no longer permitted to members of the profession. Possibly the change may lead to individual cases of hardship; but, even if so, it will be productive of public good, and those who suffer will have been abundantly forewarned.

